# Alveolar Hemorrhage Caused by the Combination of Immune Checkpoint Inhibitors (ICIs) and Angiogenesis Inhibitors: The Underlying Long-Term Vascular Endothelial Growth Factor (VEGF) Inhibition

**DOI:** 10.7759/cureus.23272

**Published:** 2022-03-17

**Authors:** Naoki Shijubou, Takeyuki Sawai, Taku Hatakeyama, Satoru Munakata, Masami Yamazoe

**Affiliations:** 1 Respiratory Medicine, Hakodate Municipal Hospital, Hakodate, JPN; 2 Pathology, Hakodate Municipal Hospital, Hakodate, JPN

**Keywords:** long-term vegf inhibition, adverse events, diffuse alveolar hemorrhage, combination therapy, anti-angiogenesis inhibitor, immune checkpoint inhibitor

## Abstract

The combination of immune checkpoint inhibitors (ICIs) and other anticancer agents is the standard of care for various cancers. Bevacizumab, an anti-angiogenesis inhibitor, causes serious adverse events such as pulmonary hemorrhage (PH). Here, we present a case of drug-induced diffuse alveolar hemorrhage (DAH), an adverse event, in a patient with hepatocellular carcinoma who was treated with a combination of ICIs and anti-angiogenesis inhibitors after long-term use of lenvatinib, which inhibits vascular endothelial growth factor (VEGF). An 85-year-old man with hepatocellular carcinoma initially received lenvatinib, a multi-kinase inhibitor, but the drug was later switched to bevacizumab-atezolizumab combination therapy owing to disease progression. After five cycles, he developed dyspnea and diffuse ground-glass opacities, which improved with discontinuation of the combination therapy and initiation of steroid pulse therapy. Our case findings indicate that both ICIs and anti-angiogenesis inhibitors cause drug-induced DAH, and their combination may increase the severity of DAH. Moreover, long-term VEGF inhibition may induce the development of DAH. Clinicians need to be aware that long-term VEGF inhibition may be associated with DAH and should consider the risk management of such adverse events while using this combination therapy.

## Introduction

Recently, the use of immune checkpoint inhibitors (ICIs) together with other anticancer agents (e.g., cytotoxic anticancer agents, angiogenesis inhibitors) has become the standard of care for various cancers [1-4]. Atezolizumab, an ICI, interferes with the binding of programmed cell death 1 ligand 1 (PD-L1) to its two receptors, programmed cell death 1 (PD-1) and B7.1 [5]. Atezolizumab inhibits immunosuppressive signals by blocking the PD-L1/PD-1 immune checkpoint within the tumor microenvironment and consequently, increases T-cell-mediated immunity against the tumor [5]. Adverse events associated with the use of ICIs include lung failure, particularly diffuse alveolar hemorrhage (DAH), which can sometimes be fatal. Therefore, it is important to manage these life-threatening conditions [6,7].

Pulmonary hemorrhage (PH) is a serious adverse event associated with bevacizumab, a monoclonal antibody that targets vascular endothelial growth factor (VEGF) [8]. Tumor infiltration into the mediastinum and major vessels and tumor cavitation in the lung can be the underlying causes for the occurrence of PH [8]. Treatment with bevacizumab causes alveolar hemorrhage even in the absence of mediastinal invasion, macrovascular invasion, or tumor vacuolation. Alveolar hemorrhage may occur because of fragile pulmonary capillary walls, resulting from VEGF inhibition [9,10]. Lenvatinib is a multi-kinase inhibitor that inhibits VEGF.

Herein, we report a case of alveolar hemorrhage that occurred as an adverse effect of the concomitant use of an angiogenesis inhibitor and ICI after the long-term use of lenvatinib.

## Case presentation

An 85-year-old man regularly visited our hospital for chronic hepatitis C treatment since 2004. There was no indication of respiratory disease, but he had a smoking history of 40 pack-years. In 2011, he was diagnosed with hepatocellular carcinoma T2N0M0 Stage II after undergoing partial hepatectomy to resect a mass in hepatic segments 5 and 8. In 2016, the mass recurred in hepatic segment 8, and he, therefore, underwent radiofrequency ablation. In September 2019, the tumor recurred in hepatic segment 8. Therefore, treatment with lenvatinib, a multi-kinase inhibitor, was started. In September 2020, trans-catheter arterial chemoembolization was performed.

**Figure 1:**
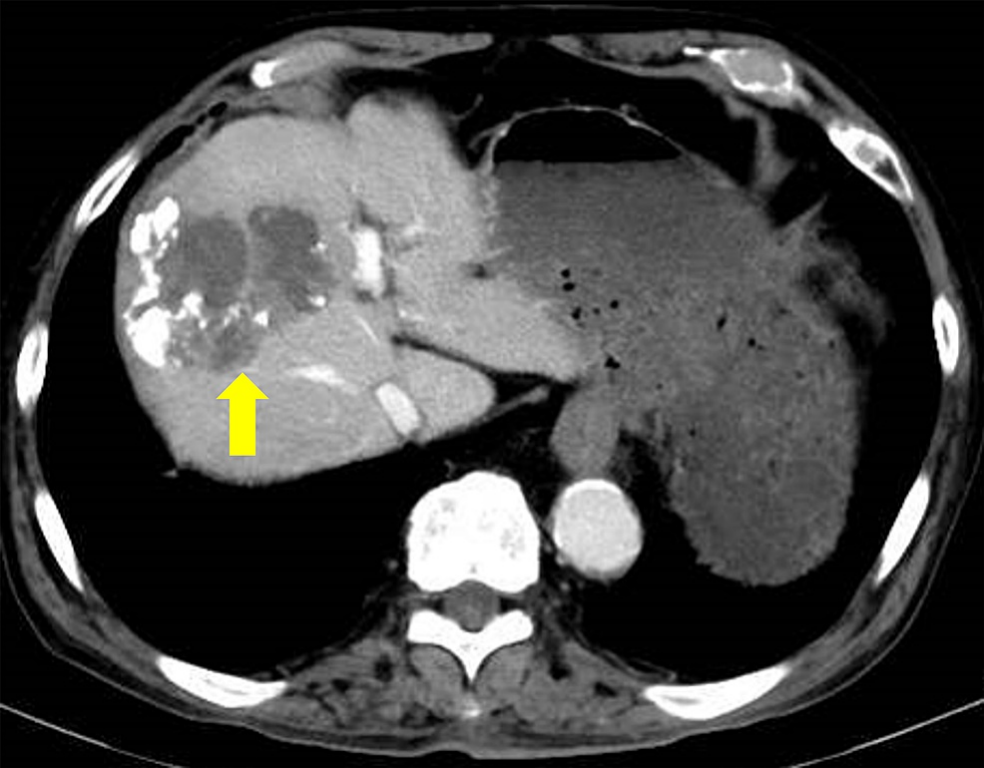
Abdominal computed tomography findings Abdominal computed tomography findings of hepatocellular carcinoma. An enlarged mass was then found in hepatic segment 8 (arrow).

**Figure 2:**
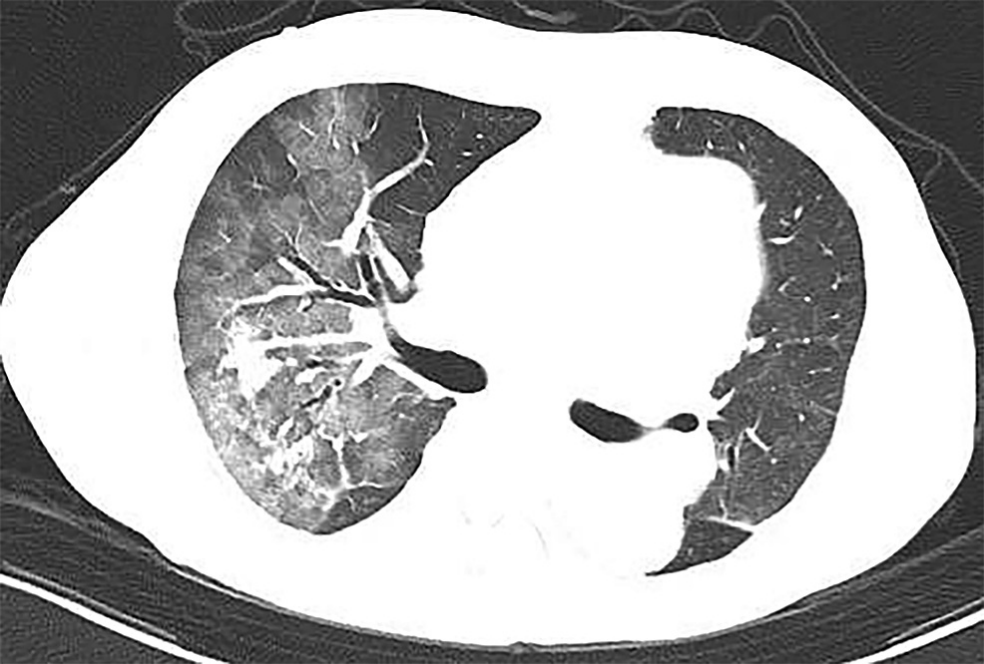
Chest computed tomography findings on admission Chest computed tomography revealed the presence of diffuse ground-glass opacities.

Lenvatinib was continued until January 2021. Owing to the disease's progression, the drug was switched to bevacizumab-atezolizumab combination therapy. Thereafter, an enlarged mass was found in hepatic segment 8 (Figure 1). The best response to the combination therapy was stable disease. However, after receiving five cycles of the combination therapy within one week, the patient developed dyspnea. Chest computed tomography revealed the presence of diffuse ground-glass opacities (Figure 2). On admission, his oxygen saturation was 92%, which was achieved with a mask providing 5 L oxygen/min, and his respiratory rate was 24 breaths/min. Respiratory function test could not be performed due to his poor respiratory condition.

**Figure 3:**
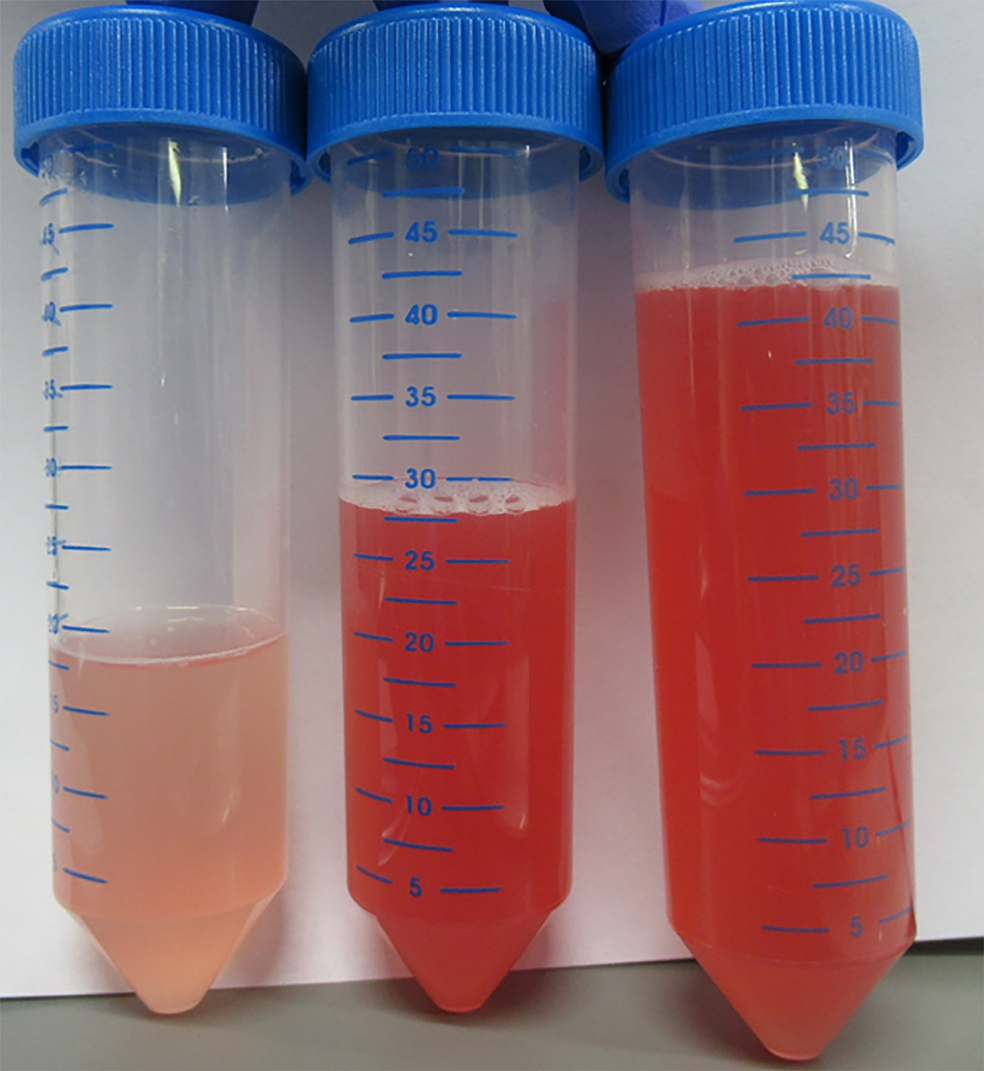
Image showing bronchoalveolar lavage (BAL) Image showing bronchoalveolar lavage (BAL) performed via the right B3 segment; blood-stained lavage fluid was observed.

Echocardiography revealed normal cardiac function. The polymerase chain reaction (PCR) analysis results for coronavirus disease-19 (COVID-19), using nasal swab fluid, was negative. The prothrombin time was 16.1 (reference value: 10.5-13.5) s, and the D-dimer level was 2.2 (reference value: <1.0) μg/mL. Only mild coagulation abnormalities were noted. The Krebs von den Lunge-6 and surfactant protein-D levels were within the normal limits. The levels of antinuclear antibody, proteinase-3 anti-neutrophil cytoplasmic antibodies (ANCA), myeloperoxidase ANCA, and anti-glomerular basement membrane antibodies were also within the normal limits. Bronchoalveolar lavage (BAL) was performed in segment 3 of the right upper segment bronchus, and the BAL fluid (BALF) was found to contain blood (Figure 3).

**Figure 4:**
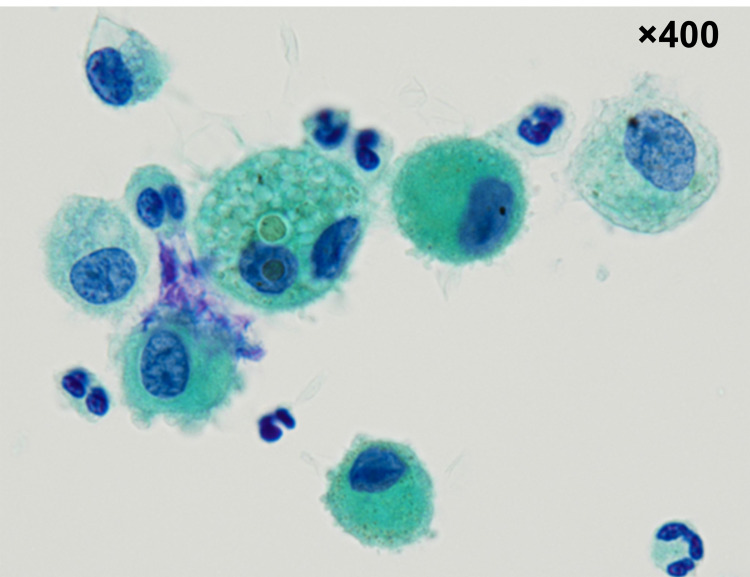
Cytological examination of the bronchoalveolar lavage fluid (BALF) Cytological examination of the bronchoalveolar lavage fluid (BALF) using Papanicolaou-stained smears showing hemosiderin-laden macrophages.

An analysis of the BALF cell count revealed neutrophilic inflammation (64% of the volume recovered; total cell count: 7.6 × 105 cells/mL, neutrophil count: 70.4%, eosinophil count: 0%, lymphocyte count: 4.6%, macrophage count: 25%). Cytological examination showed no malignant cells, fungal elements, or viral cytopathic changes; only hemosiderin-phagocytosing macrophages were observed (Figure 4). There were no notable bacterial, mycobacterial, or fungal pathogens in the BALF cultures. A PCR analysis of BALF showed no *Pneumocystis jiroveci i*nfection.

Based on the BALF findings and the exclusion of diseases causing DAH, we diagnosed the patient with drug-induced DAH. Therefore, the combination therapy was discontinued, and steroid pulse therapy (methylprednisolone, 1 g daily) was initiated, after which the ground-glass opacities improved on Day 4 of admission.

## Discussion

We report a case of alveolar hemorrhage that occurred as an adverse effect of the concomitant use of an angiogenesis inhibitor and ICI after the long-term use of lenvatinib, which inhibits VEGF. The condition improved on discontinuing the combination therapy and initiating steroid pulse therapy.

DAH may be caused by various diseases, such as congestive heart failure, infections, coagulation disorders, collagen diseases, and vasculitis [10]. Most cases of DAH are due to the breakdown of the alveolar capillary barrier resulting from immunological factors, such as vasculitis or specific drug reactions [11]. Here, the patient was diagnosed with drug-induced DAH by excluding other diseases causing DAH. The mechanism underlying the pathogenesis of DAH-associated immune-related adverse events is not fully understood, but it is hypothesized that cytotoxic T lymphocytes damaged endothelial cells in the small blood vessels of the lung [6].

In the present case, although the patient was diagnosed with DAH, we could not determine whether it was caused by ICIs or angiogenesis inhibitors. Most cases of DAH are considered to be caused by the disruption of the alveolar capillary wall [12]. The VEGF not only enhances endothelial cell proliferation but is also important for maintaining vascular integrity; VEGF inhibition by bevacizumab administration may increase the friability of the capillaries [13].　Lenvatinib is a multi-kinase inhibitor that inhibits VEGF and, having been administered before the combination therapy and the underlying long-term VEGF inhibition, may have been associated with DAH.

Additionally, PD-1 and PD-L1 inhibitors may cause hemorrhagic complications due to immune activation, which may disrupt the balance of the coagulation-fibrinolysis system [14]. Although risk factors for predicting the development of DAH have not been identified, older age and smoking history are known risk factors for the development of acute lung injury and acute respiratory distress syndrome [15,16]. Our patient was elderly and had a history of smoking, which could have contributed to the development of DAH.

## Conclusions

Long-term VEGF inhibition may increase the friability of the capillaries. Thus, clinicians need to be aware that long-term VEGF inhibition may be associated with DAH. The combined use of angiogenesis inhibitors and ICIs could increase the severity of alveolar hemorrhage. Therefore, clinicians should pay more attention to the management of adverse effects when such a combination therapy is administered.
